# Ileo-Anal Pouch Anastomosis and New Remedial Approaches for Ulcerative Colitis: A Review Article

**DOI:** 10.7759/cureus.34027

**Published:** 2023-01-21

**Authors:** Abhijeet Jankar, Tripti Shrivastava

**Affiliations:** 1 Physiology, Jawaharlal Nehru Medical College, Datta Meghe Institute of Medical Sciences, Wardha, IND

**Keywords:** ipaa, surgery, protocolectomy, pouchitis, ulcerative colitis

## Abstract

Ulcerative colitis (UC) causes diffuse friability and superficial wall degeneration that is accompanied by bleeding. UC, now recognized as a global illness, affects millions of people globally. The most effective treatment for UC is Ileal Pouch Anal Anastomosis (IPAA). Many medical experts and patients favor proctocolectomy with IPAA because it improves bowel function and allows feces to pass via the anus. Considering the most recent research, systematic reviews, statistical analysis, and recommendations, a selective literature search was carried out. The database used was PubMed. The current work provides a summary of surgical alternatives, results, and pre-and postoperative treatment for UC patients. In the course of their illness, about 30% of UC patients will need surgery. Due to its natural limitation to the colon and rectum, UC may mostly be treated surgically. The preferred surgical process is a restorative proctocolectomy with an IPAA. A 30% postoperative complication rate and a 0.1% death rate for this operation are both shown in large studies. One of the biggest things preventing UC from being successfully treated surgically is pouchitis. A long-run success rate of the pouch is >90% after 10 follow-ups, despite a significant surgical complication rate. For patients with UC to have the best possible outcome, extensive collaboration among the various specialties in the pre and postoperative context is crucial. In skilled centers, more than 90% of the total patients can eventually achieve a decent quality of life despite a 30% likelihood of early postoperative problems. UC patients may be cured with proctocolectomy, but there is a risk of morbidity that must be considered, especially in pediatric patients. As a result of advancements in our comprehension of the pathogenic mechanisms causing UC, new therapies have been proposed, the most significant change being the emergence of anti-tumor necrosis factor (TNF) medications.

## Introduction and background

An idiopathic inflammatory disorder of the colon known as UC (Ulcerative Colitis) causes diffuse friability and superficial degradations on the colonic wall that are accompanied by hemorrhage. UC is a chronic illness that can manifest at different phases of the illness's severity and activity. Typically, it only affects the colon's mucosa and submucosa, causing inflammation [1]. Millions of individuals worldwide suffer from ulcerative colitis, which is now regarded as a global illness [2]. Blood in the stool, severe diarrhea, and frequent rectal bleeding are all symptoms of UC. Additionally, 50% of UC patients have an iron shortage [3]. Certain food ingredients have a significant impact on how UC develops by altering the composition and functionality of the gut microbiota. The range of available treatments for ulcerative colitis has expanded quickly and will do so in the future. None of the medications that are currently available or being developed will either treat the illness or even target the underlying pathology. Additionally, no medicine that attains and sustains recovery in most patients is currently on the market or close to it. Although having more options is a big improvement for those who had previously failed treatment, UC still has no effective medical treatment outside of surgery [4]. The colon and rectum can be surgically removed to treat UC. IPAA is the best method for treating UC [5]. Up to 30% of ulcerative colitis individuals will need surgery at some point during their condition [6]. UC surgery is needed in several cases: severe intestinal inflammation nonresponsive to therapeutic treatment; disease later refractory to medical care; patients unable to endure medical care or its side effects and cases of tumoral or premalignant transformation of the inner lining of the intestinal tract [7]. The only treatment (restorative proctocolectomy with IPAA) completely removes the chances of both inflammation and cancer in the colonic and rectal regions. To prevent anastomotic leaking, the difficult IPAA operation is often carried out in two or three phases in instances of colonic inflammatory bowel disease (IBD) [8]. Dysplasia and pouchitis were the most frequent side effects of restorative proctocolectomy. Pouchitis was the most typical complication among UC patients who underwent surgery [9]. One of the most efficient medical procedures to address some serious health conditions is surgery. Laparoscopic surgery is a cutting-edge surgical technique that claims to address some underlying abdominal or pelvic health conditions more quickly and painlessly. With just a little incision into the body, surgeons can fix the issue with previously unheard-of simplicity and perfection while having a great view of the internal structure. Small scars, blood loss, less discomfort, shorter hospital stays, lower risk of infections, and quicker recovery are just a few of the many advantages of laparoscopic surgery. Due to their lengthier operating periods and intrinsic technical difficulty, complex laparoscopic colorectal resections have historically been criticized. The specialty of complicated laparoscopic surgery, including the IPAA operation, has evolved thanks to technological advancements and increasing surgical proficiency with laparoscopy, with safe achievable outcomes. The intricacy of the laparoscopic ileal pouch-anal anastomosis (IPAA) technique is simplified when these procedures are divided into manageable steps, making it possible to successfully repeat this operation. The sequential laparoscopic steps described here create a straightforward, consistent method for performing laparoscopic IPAA surgery on patients with UC. One has a practical approach to this challenging operation with its laparoscopic IPAA technique [10]. For restorative proctocolectomy, IPAA is a safe procedure with a low risk of morbidity. Patient satisfaction is high, and functional outcomes are typically positive [11]. Because proctocolectomy with IPAA restores bowel function and allows feces to flow via the anus, this surgical procedure is preferred by many healthcare professionals and patients.

## Review

Methodologies

**Figure 1:**
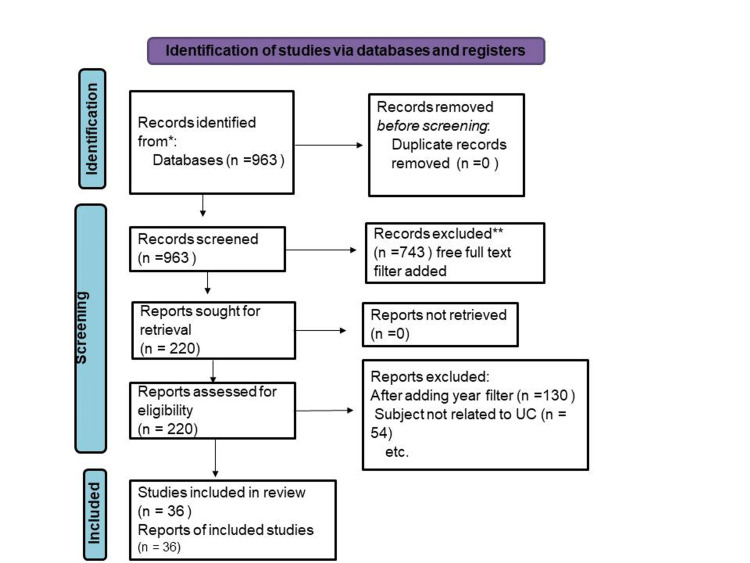
PRISMA flow diagram for ileo-anal pouch anastomosis and ulcerative colitis. *Consider, if feasible to do so, reporting the number of records identified from each database; ** If ambulation tools were used, indicate how many records were excluded by a human and how many were excluded by automation tools.

We undertook a systematic search through PubMed and CENTRAL in August 2022 using keywords such as “Ileo-Anal Pouch Anastomosis” and “Ulcerative Colitis” (((Ileo-Anal Pouch Anastomosis [Title/Abstract]) OR (IPAA [Title/Abstract])) OR (“Ileo-Anal Pouch Anastomosis” [MeSH Terms]) AND ((“Ulcerative Colitis” [Title/Abstract]) OR (UC [Title/Abstract])) OR (“Ulcerative Colitis” [MeSH Terms]). We additionally searched for key references from bibliographies of the relevant studies. The search was updated in February 2020. One reviewer independently monitored the retrieved studies against the inclusion criteria in the beginning, based on the title and abstract and then on full texts. Another reviewer also reviewed approximately 20% of these studies to validate the inclusion of studies. Differences were resolved through discussion. One reviewer extracted the data from studies according to the mental, physical, and social health aspects of UC. Data were extracted and tabulated on details about the study design, population, UC ileo-anal pouch anastomosis instrument used, UC domain assessed, and study ﬁndings. We included studies that assessed the effect of ileo-anal pouch anastomosis on UC both during and after treatment, irrespective of the study design, geographic location, age, gender, or type of treatment. For inclusion, both published and unpublished studies in the English language were considered. We excluded studies that were published in other languages because of resource limitations or if the full-text articles were unavailable to reviewers. We also excluded studies other than ileo-anal pouch anastomosis, or ileo-anal pouch anastomosis has been considered as one of the comorbidities along with any other chronic conditions or UC was not assessed, as shown in Figure 1. 

Exclusion criteria: After adding the year filter (n= 130), the subject was not related to ileo-anal pouch anastomosis and UC (n=54).

Inclusion criteria: Free full-text articles included according to ileo-anal pouch anastomosis and ulcerative colitis, the studies included in the review (n=36), and reports of included studies (n=36).

Indications for emergency surgery

With diarrhea and bleeding at first, UC progressively becomes worse, but it can also advance quickly to the point where immediate surgery is necessary. The reevaluation of surgery's function in illness management, with a focus on the value of multidisciplinary decision-making in challenging circumstances [12]. Patients with UC experience serious hemorrhage at a rate of 4.5% or less frequently. The requirement for more than four red cell concentrates every 24 hours or for a patient who experiences severe initial hemorrhage, hemodynamic instability, and the need for catecholamines are both indications for surgery. An interdisciplinary approach is taken to figure out the cause of a persistent sudden flare [13]. A rare but possibly dangerous side effect of fulminant colitis is toxic megacolon. Inflammatory bowel disease patients have a higher likelihood of experiencing toxic colonic dilation, which can also be brought on by ischemic or pathogenic inflammation, such as pseudomembranous colitis. A toxic mega-colon is characterized by regional or entire colon enlargement of >6 cm in addition to the presence of toxicity and severe colitis clinical symptoms [14]. If a significant improvement is not achieved through acute medical treatment combined with targeted anti-inflammatory medical therapy over 72 hours, the persistent sudden flare offers immediate surgery. Recognizing an unintentional escalation of sepsis is difficult for surgeons because the disease's clinical symptoms are frequently controlled by high-dose corticosteroids and antibiotics [13]. A patient undergoing radiation treatment for prostate cancer who developed UC. Rapid UC progression necessitated immediate surgery [15].

Methods

A one-stage procedure is a proctocolectomy with IPAA performed without a protective ileostomy. The typical two-stage surgery consists of proctocolectomy with IPAA and loop ileostomy, then ileostomy closure. Modified two-stage procedure: a partial colectomy with an end ileostomy was followed by a proctectomy with IPAA without a protected ileostomy. A three-stage procedure consists of a proctectomy with IPAA and a protected ileostomy, followed by a partial colectomy with an end ileostomy, a protected ileostomy, and ileostomy closure [16]. Surgery for UC patients who need it is IPAA. W, S, or J ileal pouch is anastomosed either using a hand-sewn or stapled approach in IPAA. It was initially defined as a hand-sewn anastomosis and an S-pouch combined. Although a pelvic pouch is simpler to make and a stapled intestinal anastomosis is linked to operational outcomes than a hand-sewn IPAA, these techniques have been widely used. Infrequently, the maintenance of an intermediate region for a stapled IPAA results in the growth of a malignant tumor [17].

The most common complication after IPAA: pouchitis

Pouchitis was the most typical consequence after IPAA in UC patients (49%). The overall pro-inflammatory characteristics of UC are most likely responsible for the frequency of pouchitis that occurs after IPAA for UC [18]. Common symptoms of pouchitis include increased bowel movements, watery stools, stomach pains, tenesmus, incontinence, and pelvic pressure [19]. Treatments for pouchitis are antibiotics (drugs for bacterial infections), budesonide enemas, probiotics (beneficial bacteria), biologicals that target tumor necrosis factor, butyrate suppositories, glutamine suppositories, bismuth enemas (diarrhea medicine), allopurinol and tinidazole [20]. The most often prescribed medications in a review of pouchitis treatments were ciprofloxacin (seventy percent), metronidazole (sixty percent), and amoxicillin-clavulanate (twenty percent). The two antibiotics that were most frequently administered as a first line of treatment for pouchitis were ciprofloxacin and metronidazole [21]*.*

**Figure 2:**
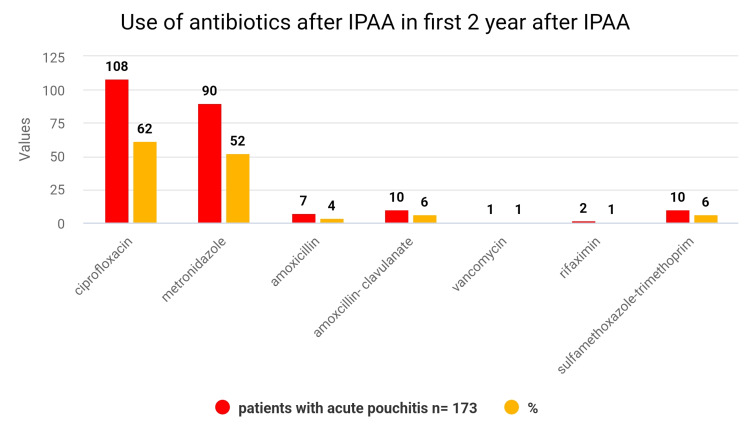
Use of antibiotics after IPAA in the first 2 years after IPAA: acute pouchitis [21]

**Figure 3:**
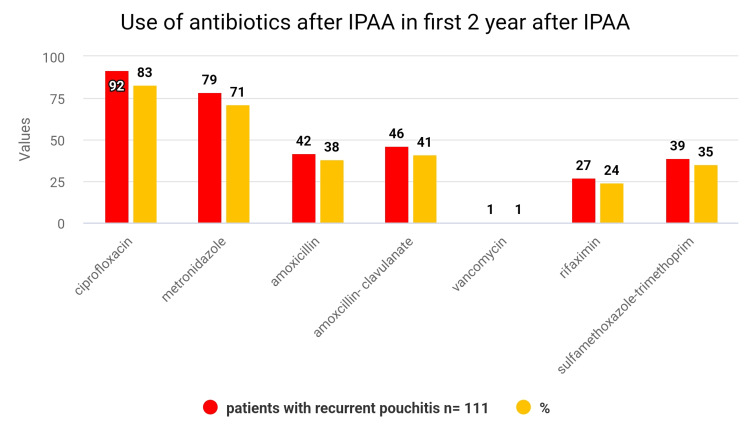
Use of antibiotics after IPAA in the first 2 years after IPAA: recurrent pouchitis [21]

When compared with severe pouchitis patients, those with recurrent patients of pouchitis showed greater use of ciprofloxacin, metronidazole, and amoxicillin-clavulanate antibiotic prescriptions. Additionally, in the two years following IPAA, recurrent pouchitis patients had a higher average value of antibiotic medication fills. Those who experienced pouchitis were contrasted with those who did not, or when trying to compare recurrent pouchitis patients to those with severe pouchitis, there was no higher probability of growing Clostridioides difficile inflammation in the two years following IPAA above (Figures 2, 3) [21].

Postoperative complications after IPAA

Fecal incontinence is a frequent problem associated with IPAA, and it gets worse over time. Age at the time of surgery, a longer period since the onset of the disease, being female, and having a lower preoperative maximum anal squeezing pressure are risk variables associated with fecal incontinence in IPAA patients, and these characteristics may alter the outcome of sacral nerve stimulation therapy [22]. Small intestinal obstruction, pouchitis, and perianal fistula were significant late postoperative complications in pediatric patients, with surgical site infection and small bowel obstruction being the most frequent early postoperative issues. Pouchitis was the most frequent late surgical complication, with a pelvic pouch survival rate of 91.7% following 10 years. Regarding the cumulative pouch survival rate, there were no notable gender or anastomotic technique differences. Proctocolectomy may provide UC patients with a cure, but there is a risk of morbidity that must be taken into account, particularly in pediatric patients. It is vital to be aware that an anal fistula and pouchitis may result in pouch failure in pediatric patients with UC, which should be prevented after a restorative proctocolectomy [23]. Proctitis is frequently present during IPAA. Poor long-term outcomes are linked to high levels of inflammation; however, this effect fades over time. Additionally, a higher proctitis level causes pouch failure to occur earlier [24].

Quality of life after IPAA

Six months following surgery, proctectomy had no impact on the sexual function of patients with UC. No matter the age, type of procedure, or ailment, this result remained constant. In contrast to men, female patients appear to experience poor sexual function more commonly, despite proctectomy having no impact on either sex's sexual life. For UC patients needing proctectomy, careful nerve-sparing rectal dissection by knowledgeable colorectal surgeons should be the cornerstone [25]. Colectomy patients have a greater overall standard of living than the general population. The most frequent adverse effects following colectomy were impotence and incontinence, whereas rectal discharge and the frequency of defecation per day without the application of anti-diarrheal medications were adverse effects that severely reduced the standard of living [26].

Morbidity and mortality

In skilled hands, the IPAA is a difficult operational technique with reasonable rates of morbidity but minimal fatality. Numerous investigations have revealed that postoperative complications have a rate of mortality is 0.1% and occur at a rate of about 30%. In 95% of patients, operational outcomes and standard of living were outstanding or excellent, and these results were consistent across all histological subgroups [27].

Preventing, monitoring, and managing IPAA metabolic complications

A risk factor of iron deficiency anemia exists in individuals with healthy and inflammatory IPAAs [28]. After pouch surgery, roughly one-quarter of patients had subnormal vitamin B12 levels. Uncertainty surrounds the precise mechanism of B12 insufficiency in these people. Sequential measurements of vitamin B12 levels show a decline in the majority of restorative proctocolectomy patients. Following treatment with oral B12 replacement medication, patients with low serum B12 levels should successfully see their levels return to a normal value. Levels of serum B12 should be tested during the investigation [29]. Independent of pouch inflammation, IPAA patients also had a greater frequency of deficiency of vitamin D and serum calcium levels. 10% to 68% of patients are vitamin D deficient. In IPAA, bone loss is typical. Age, pouchitis, low body mass index (BMI), pouch villous atrophy, primary sclerosing cholangitis, and a lack of calcium supplementation are all risk factors. For all patients, we advise a basal bone mineral density measurement. We also advise calcium and vitamin supplements in people who have osteopenia-related risk factors such as low vitamin D or calcium levels.

Diagnostic tests for IPAA evaluation

Pouchoscopy: This procedure can be carried out using either a gastroscope or a colonoscope, though we favor the former. The pouch body, prepouch ileum, and cuff are the three sections that need to be examined. Retroflexion is important if a fistula is suspected and valuable for evaluating the rectal cuff. The three regions that were inspected should have biopsies performed, with suture lines being avoided.

Imaging: CT scan or MRI of the pelvis is most useful for examining potential perianal or peripouch problems as well as early and later mechanical or surgical issues. When examining problems connected to obstructive pouches, barium defecography is helpful [28].

The impact of modern drug therapy on surgery: UC

The number of medicines that have been approved will increase as conservative management for UC treatment continues to advance. For some patients, surgery is a good option for protracted conservative therapy rather than the detrimental goal of treatment approaches. To maximize the timing and result of UC patients, extensive collaboration across the many specialties in pre- and postoperative therapy is crucial [30].

The continuation of medical therapy or elective surgery?

The majority of UC patients won't require surgery because of the efficacy of contemporary medical care. There is a subset of individuals with treatment-refractory illness, however, which will unquestionably need an operation in the long run. Only a temporary improvement can be shown in those individuals from biological therapy, delaying the requirement for a colectomy. Patients who have a chronic illness, poor drug adherence, or cancer discomfort will significantly benefit from surgery, in addition to those who are unresponsive to pharmacological treatment [31]. The goal of a restorative proctocolectomy is to eliminate the disease's source and restore gastrointestinal continuity. After IPAA, the majority of patients are able to avoid hospital stays and colitis-related drugs, including immunosuppressant drugs and immunomodulatory, and any potential side effects they may have. The surgery also significantly lowers the symptoms of UC and the risk of dysplasia or malignancy [13]. There was no variation in the standard of living between patients with pouches and those receiving anti-tumor necrosis factor (TNF) medication. However, patients having pouches had a better standard of living ratings and were paid less overall for medical treatment [32]. Therefore, compared to long-term pharmacological therapy, surgery is a good choice. Approximately 90% of patients can eventually gain a satisfactory standard of living, despite the issues that can arise after IPAA.

New remedial approaches

New treatments have been suggested as a result of improvements in our comprehension of UC pathogenicity, with the emergence of anti-TNF medications ranking as the most significant breakthrough [33]. For the treatment of UC, numerous novel compounds are currently being tested, such as anti-mucosal cell adhesion molecule 1 agent, Janus kinase inhibitors, anti-interleukin-12/23 monoclonal antibodies, anti-SMAD7 antisense oligonucleotides, and sphingosine-1-phosphate receptor-1 specific agonists. By altering the course of the disease, anti-TNF medications (infliximab, certolizumab, and adalimumab) have decreased the necessity for operation and hospitalization and enhanced patients' standard of living. Thus, recommendations make anti-TNF medicines the first line of therapy for moderate-to-severe UC or if non-biological therapy is unsuccessful. Patients who originally reacted to therapy have also abandoned their responsiveness over time; therefore, these therapies have not been successful in all patients [34]. An anti-inflammatory medication without side effects, such as not increasing the risk of postoperative complications, would be ideal from a surgical perspective. Intestinal alkaline phosphatase, a gut brush border enzyme, works as an anti-inflammatory epithelial defense component that detoxifies several pro-inflammatory derivatives, including lipopolysaccharides and CpG-DNA, and supports the integrity of the intestinal barrier [35]. Intestinal alkaline phosphatase mRNA levels are decreased in UC patients [36]. To further assess this enzyme's potential as an efficient and well-tolerated treatment for UC, however, an acid-resistant oral version is required.

## Conclusions

Proctocolectomy may provide UC patients with a cure, but there is a risk of morbidity that must be taken into account, particularly in pediatric patients. Six months following surgery, proctectomy had no impact on the sexual function of patients with UC. Sequential measurements of vitamin B12 levels show a decline in the majority of restorative proctocolectomy patients. After IPAA, the majority of patients are able to avoid hospital stays and colitis-related drugs, including immunosuppressant drugs and immunomodulatory, and any potential side effects they may have. The surgery also significantly lowers the symptoms of UC and the risk of dysplasia or malignancy. After IPAA, the majority of patients are able to avoid hospital stays and colitis-related drugs, including immunosuppressant drugs and immunomodulatory, and any potential side effects they may have. The surgery also significantly lowers the symptoms of UC and the risk of dysplasia or malignancy. After IPAA, the majority of patients are able to avoid hospital stays and colitis-related drugs, including immunosuppressant drugs and immunomodulatory, and any potential side effects they may have. The surgery also significantly lowers the symptoms of UC and the risk of dysplasia or malignancy. Patients having pouches had a better standard of living rating and paid less overall for medical treatment. Approximately 90% of patients can eventually gain a satisfactory quality of life, despite the issues that can arise after IPAA. UC patients may be cured with proctocolectomy, but there is a risk of morbidity that must be considered, especially in pediatric patients. As a result of advancements in our comprehension of the pathogenic mechanisms causing UC, new therapies have been proposed, with the launching of anti-TNF drugs ranking as the most important development.
